# Macrophage Migration and Invasion Is Regulated by MMP10 Expression

**DOI:** 10.1371/journal.pone.0063555

**Published:** 2013-05-14

**Authors:** Megan Y. Murray, Timothy P. Birkland, Jonathan D. Howe, Andrew D. Rowan, Mark Fidock, William C. Parks, Jelena Gavrilovic

**Affiliations:** 1 School of Biological Sciences, University of East Anglia, Norwich Research Park, Norwich, Norfolk, United Kingdom; 2 Center for Lung Biology, University of Washington, Seattle, Washington, United States of America; 3 Pfizer Global Research and Development, Sandwich, Kent, United Kingdom; 4 Musculoskeletal Research Group, Institute of Cellular Medicine, The Medical School, Newcastle University, Newcastle, United Kingdom; South Texas Veterans Health Care System and University of Texas Health Science Center at San Antonio, United States of America

## Abstract

This study was designed to identify metalloproteinase determinants of macrophage migration and led to the specific hypothesis that matrix metalloproteinase 10 (MMP10/stromelysin-2) facilitates macrophage migration. We first profiled expression of all MMPs in LPS-stimulated primary murine bone marrow-derived macrophages and Raw264.7 cells and found that MMP10 was stimulated early (3 h) and down-regulated later (24 h). Based on this pattern of expression, we speculated that MMP10 plays a role in macrophage responses, such as migration. Indeed, using time lapse microscopy, we found that RNAi silencing of MMP10 in primary macrophages resulted in markedly reduced migration, which was reversed with exogenous active MMP10 protein. *Mmp10* ^−/−^ bone marrow-derived macrophages displayed significantly reduced migration over a two-dimensional fibronectin matrix. Invasion of primary wild-type macrophages into Matrigel supplemented with fibronectin was also markedly impaired in *Mmp10* ^−/−^ cells. MMP10 expression in macrophages thus emerges as an important moderator of cell migration and invasion. These findings support the hypothesis that MMP10 promotes macrophage movement and may have implications in understanding the control of macrophages in several pathologies, including the abnormal wound healing response associated with pro-inflammatory conditions.

## Introduction

Matrix metalloproteinases (MMPs) are a family of highly conserved, zinc-dependent endopeptidases with a range of functions in immunity, tissue repair and other disease processes. [1], [2]. In healthy tissue the activity of MMPs is tightly regulated with a delicate balance between activation and inhibition, which is mediated largely by the endogenous tissue inhibitors of metalloproteinases (TIMPs). Once activated, MMPs act on a variety of extracellular proteins, including extracellular matrix (ECM) components and the ectodomains of membrane proteins. This can have consequences on cell-matrix interactions and subsequent migration, cytokine signalling, and leukocyte activation in both normal and pathological processes (reviewed in [3], [4], [5]). The broad and varied function of MMPs highlights the importance of expanding our understanding of these proteinases in disease pathology, including inflammation and impaired wound healing.

Macrophages express a number of MMPs and are key players in several repair processes and pathologies, including the wound healing process. Human macrophages and their monocytic precursors can express a range of MMPs [6], [7], [8] and this expression profile is modulated during the process of differentiation from precursor monocyte to macrophage, concomitant with morphological changes, such as cell adhesion and spreading [9], [10]. Induction of macrophage MMP expression can be driven by pro-inflammatory stimuli, such as TNFα, gram-negative lipopolysaccharide (LPS), and type II interferons [7], [8], [11].

The activation of leukocytes in response to LPS via TLR4/NF-κB signalling is well-characterised, including an important role in the transcription of some MMPs. For example, pharmacological inhibition of NF-κB translocation into the nucleus, in both primary monocytes and cell lines *in vitro*, represses the expression of several MMPs [12], [13]. *In vivo* and *ex vivo* studies have also revealed a clear relationship between LPS and MMP expression in a variety of tissues [14], [15].

The migration of macrophages, and indeed other highly motile cells, is greatly influenced by the composition of the local ECM [16], affecting both the persistence and directionality of migration *in vivo* (reviewed in [17], [18]). Advances in our understanding of proteinase-dependent cell migration/invasion have come from studies assessing expression and function of MMPs during migration on or through various matrix components that would be present in or around sites of diapedesis [19], [20], [21], [22]. In macrophages, MMP14 has been localised to the cell’s leading edge [23] and more recently around podosomes, actin-rich adhesions, in dendritic cells where it is thought to play a role in cell protrusion [24].

*In vivo* evidence supports the idea that certain MMPs may be involved both positively and negatively in monocyte/macrophage migration. Thus Johnson and colleagues [25] have shown that macrophages are dependent on TIMP2-inhibitable MMP activity for *in vivo* colonisation of atherosclerotic plaques (as well as *in vitro* migration). Similarly, double *Mmp2^−/−/^Mmp9^−/−^* monocytes from knockout mice show reduced infiltration, suggestive of a reduction in migration, across the parenchymal basement membrane in an experimental autoimmune encephalomyelitis (EAE) model [26]. Conversely, MMPs can also exert a negative influence on macrophage migration *in vivo*. For example, macrophage recruitment in lungs following *Pseudomonas aeruginosa* infection is restrained by MMP28, whilst macrophages isolated from infected *Mmp28^−/−^* mice migrate more rapidly towards relevant bronchiolar lavage components *in vitro* [7].

Certain MMPs play key roles in fine-tuning the chemokine and growth factor response, particularly during the resolution of wound healing and associated inflammation (reviewed in [27]). For example, macrophages have been shown to control the clearance and recruitment of neutrophils in wounds by secreting MMP12 to cleave and inactivate pro-neutrophil chemokines CXCL-5 and -8. Over time MMP12 further contributes to the degradation of pro-monocyte/macrophage chemokines CCL2, 7, 8 and 13, disrupting their own recruitment to bring about the resolution of inflammation [4].

Given the implication of MMP expression in inflammatory pathologies we have taken an unbiased approach to determine murine macrophage expression of all MMPs following LPS stimulation. These studies reveal differential and time-dependent modulation of MMP10 expression in response to LPS. Further experiments revealed a novel role for MMP10 in macrophage migration and invasion.

## Materials and Methods

### Materials

Unless otherwise stated all chemical reagents were purchased from Sigma-Aldrich Corp. (St. Louis, MO, USA), all tissue culture reagents from Gibco Invitrogen Corp. (Paisley, Scotland, UK) and all tissue culture plastics from Nunc Thermo Fisher Scientific (Rochester, NY, USA).

### Bone Marrow-derived Macrophages

Bone Marrow-derived Macrophages (BMDM) were isolated from the femurs and tibias of C57Bl/6 mice (according to institutional guidelines and UK Home Office requirements) essentially as previously described [28]. Briefly, bone marrow was flushed from the bone cavity with a 21 g needle and syringe (BD, Oxford, UK) containing macrophage medium consisting of Roswell Park Memorial Institute (RPMI) 1640 liquid medium containing 100 units/ml penicillin/streptomycin antibiotic, 5 mM L-glutamine, 1% (v/v) sodium pyruvate, 0.5% (v/v) nonessential amino acids, 24 µM tissue culture grade β-mercaptoethanol, supplemented with 10% (v/v) fetal bovine serum (FBS; BioSera, East Sussex, UK) and 10% (v/v) L929-cell-conditioned medium (LCM) as a source of Colony Stimulating Factor-1 (CSF-1) [29]. Cells in the bone marrow flush were plated onto non-treated bacteriological petri dishes (BD Falcon, Oxford, UK) in macrophage medium. After three days of incubation at 37°C, 5% CO_2_, the non-adherent population was re-plated with fresh macrophage medium. The adherent population was discarded. After a further five days culture the non-adherent population was discarded, whilst remaining adherent BMDM were harvested for experimentation.

BMDM from *Mmp10^−/−^* mice [15] and wild-type littermates (protocols approved by the Institutional Animal Care and Use Committee at the University of Washington) were isolated as above.

### RNA Purification, Reverse Transcription and Quantitative Real Time – PCR

For analysis of gene expression BMDM (5×10^5^) were transferred into medium containing 0.2% FBS and exposed to 100 ng/mL γ-irradiated Lipopolysaccharide (LPS) purified from *E. coli* (0111:B4) for the duration of the experiment stated. Total RNA was purified from BMDM cell lysates using the RNeasy Minikit (Qiagen, West Sussex, UK) according to the manufacturer’s instructions and including an additional DNase 1 (Invitrogen Ltd, Paisley, UK) step. Purified mRNA (250 ng–1 µg) was reverse transcribed to complementary DNA (cDNA) using Superscript II Reverse Transcriptase (Invitrogen Ltd) according to the manufacturer’s instructions. Quantitative real-time PCR (qRT-PCR) reactions were performed using the 7500 Fast RT-PCR System and Taqman® primers and probes (Applied Biosystems, CA, USA) for murine metalloproteinases as described in [30] and [31] and QuantiTect probe PCR Master Mix (Qiagen) according to the manufacturer’s instructions. Forward and reverse primer and probe sequences for TNFα were designed using Primer Express software (Applied Biosystems; forward 5′ –AGACCCTCACACTCAGATCATCTTC–3′, reverse 5′ –CCACTTGGTGGTTTGCTACGA–3′, and probe 5′-FAM-CAAAATTCGAGTGACAAGCCTGTAGCCCA-TAMRA -3′). Steady state mRNA expression was normalized against 18 s ribosomal RNA expression using the comparative cycle threshold method (ΔΔC_T_). Statistical analysis of change in gene expression between two sets of data was performed using the two-tailed Student’s T-test on sample groups no smaller than n = 3.

### MMP10 Protein Immunostaining

BMDM (2×10^4^ cells per 13 mm glass coverslip) under indicated conditions were treated with 5 µM Monensin Sodium Salt for 3 h to block intracellular protein transport [32] and then fixed with a 4% (w/v) Paraformaldehyde solution. Cell membranes were permeablised with 0.1% (v/v) Triton X-100 and non-specific binding was blocked with 10% (v/v) normal donkey serum (DAKO, Ely, UK) before incubation with Sheep anti-MMP10 polyclonal primary antibody [33]. BMDM were washed to remove unbound primary antibody before incubation with Donkey anti-Sheep Alexa-Fluor 488 conjugated polyclonal IgG secondary antibody (Molecular Probes/Invitrogen, Paisley, UK). Before mounting with Hydromount mounting medium (National Diagnostics, GA, USA), 4′,6-diamidino-2-phenylindole (DAPI) nuclear stain was applied.

### Gene Silencing

BMDM (1.5×10^4^) were seeded onto 10 µg/mL bovine plasma fibronectin (Calbiochem/Merck, Nottingham, UK) coated plastic wells 24 h prior to transfection. 15 nM lyophilised siGENOME SMARTpool siRNA targeting mouse MMP10 (siMMP10; 5′-GAAUUGAGCCACAAGUUGA-3′, 5′-GAGAUGUUCACUUCGAUGA-3′, 5′-CCUCAGGGACCAACUUAUU-3′. Dharmacon, CO, USA) and AllStars Negative Control (5′- GGGAAGUCCUAUUCUUUAA-3′. Qiagen, West Sussex, UK) were combined with HiPerfect Transfection Reagent (Qiagen) and added to BMDM a further 24 h before time-lapse microscopy began. Where stated, 3 ng/mL recombinant human (r)MMP10 [34], was added to BMDM cultures for 6 h before time-lapse microscopy.

### 2D Cell Migration Assay and Time-lapse Microscopy

BMDM were seeded onto plastic wells (24 well plates) coated with rat tail collagen I (BD Biosciences, Oxford, UK), human plasma fibrinogen (Calbiochem/Merck) or bovine plasma fibronectin (all ECM components at 10 µg/mL). BMDM in 24 well plates (alone or transfected with siRNA for MMP10 as indicated) were transferred to a motorised stage within a controlled environment chamber, also at 37°C, 5% CO_2_. Cells were imaged every 10 minutes for 17 h with the AxioCam ICm 1 monochrome CCD camera attached to the Axiovert 200M wide field inverted light microscope using Axiovision 4.8.2 software (all Carl Zeiss Ltd, Herts, UK). Cell migration speed was determined following measurement of distance translocated by cells using ImageJ software [35] with gel-ins ‘Manual Tracking’ (F. Cordelières, Institute Curie, France) and ‘Chemotaxis and Migration Tool’ (Trapp and Horn, Ibidi GmbH, Germany).

### 3D Inverted Invasion Assay and Confocal Imaging

The 3D inverted invasion assay was performed as described previously [36] with some modifications. Briefly, 100 µL of Matrigel (BD Biosciences; stock mixed 1∶1 with ice-cold PBS and supplemented with bovine plasma fibronectin to a final concentration of 50 µg/mL), was transferred to a Transwell™ insert (8 µm pore; Corning, NY, USA) to polymerise at 37°C, 5% CO_2_. After polymerisation, Transwell™ inserts were inverted and 5×10^4^ wild-type or *Mmp10^−/−^* BMDM were applied directly to the underside of the insert filter and allowed to adhere for 2 h, before further inversion and gentle washing to remove any non-adherent cells. Finally, Transwell™ inserts were placed into a chamber containing macrophage medium whilst 100 ng/mL LPS was applied to the upper Transwell™ chamber to establish a chemotactic gradient through the Matrigel/fibronectin gel. Transwell™ cultures were incubated for 72 h to allow invasion into the gel. Cells were then stained with 4 µM Calcein-AM (Molecular Probes/Invitrogen). Confocal images of cells adherent to the filter were obtained, to confirm cell adhesion had remained constant across conditions during the experiment. These cells were then removed with a cotton swab to ensure only migrating cells were analysed. Serial confocal optical sections (20 µm) of the Matrigel/fibronectin gel were captured with a Leica TCS SP2 laser scanning confocal microscope and LCS software package (Leica Microsystems Ltd, Bucks, UK). For each experimental condition the inverted invasion assay was performed in duplicate Transwells and confocal data was collected from 3 fields of view per Transwell (6 fields of view in total). Experiments were repeated with cells independently isolated from 2 mice per genotype. Invasion data was quantified using ImageJ software (NIH) with gel-in ‘Area Calculator’ (Sergio Caballero, University of Florida, USA).

Unless otherwise stated all experiments were performed at least 3 times, with a representative experiment shown. Statistical analysis was performed using Student’s *t*-test.

## Results

### LPS Differentially Regulates the Expression of Metalloproteinases in Both BMDM and Raw264.7 Macrophages

**Figure 1:**
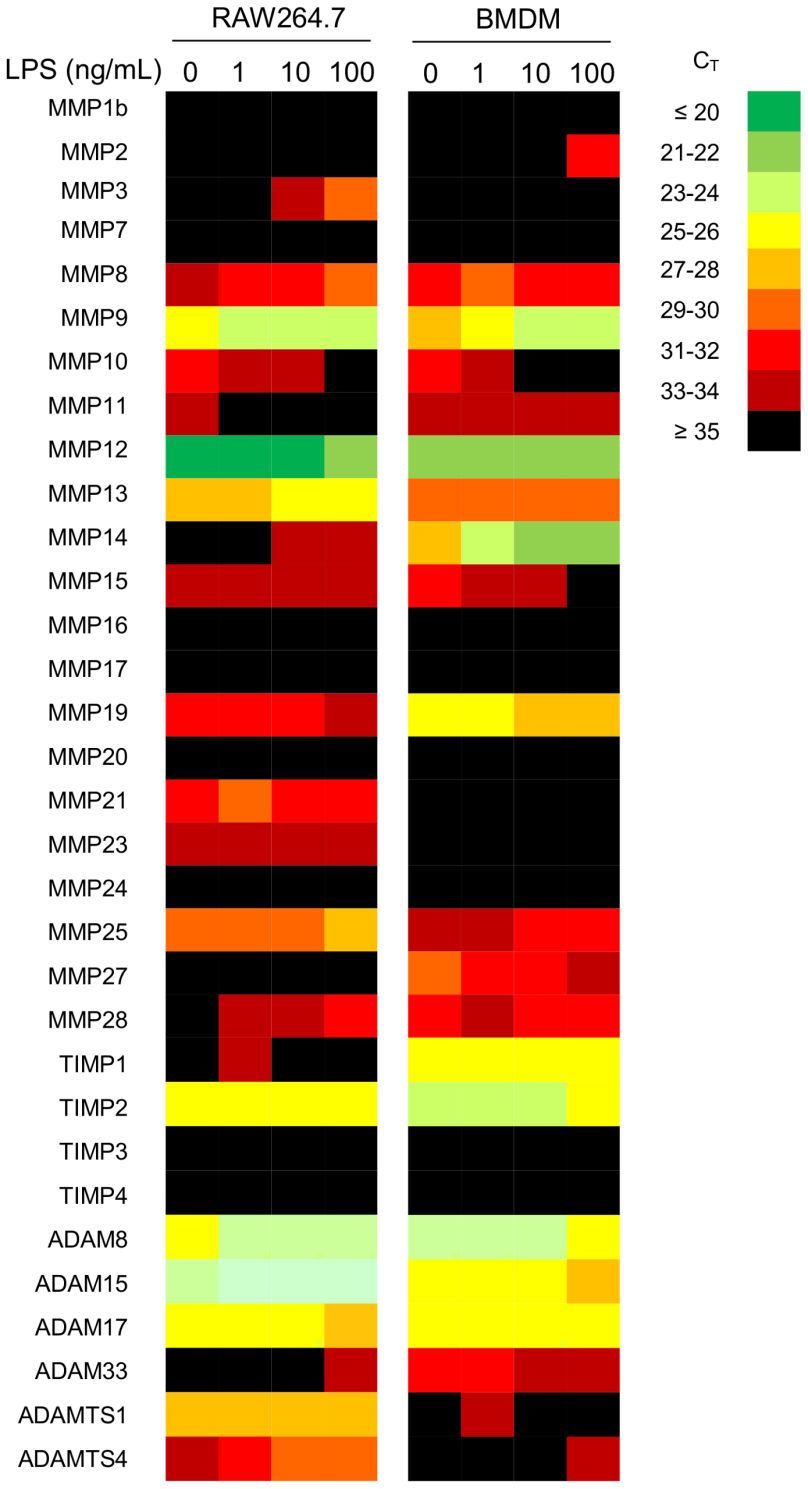
LPS regulates steady state expression of metalloproteinases and TIMPs in Raw264.7 macrophages and in BMDM. Macrophages were cultured alone or in indicated concentrations of LPS for 24 h prior to RNA extraction, reverse transcription and qRT-PCR analysis. A heat map represents the mean expression in C_T_ value for triplicate samples. The cycle threshold (C_T_) value indicates the number of PCR cycles required for the amplification of target cDNA to reach an arbitrary threshold level. A low C_T_ value indicates a greater level of target cDNA present; therefore fewer cycles are needed to reach the threshold.

Taking an unbiased approach to determine the effect of LPS on steady state mRNA metalloproteinase and TIMP expression in BMDM, qRT-PCR profiling of all MMPs, and TIMP1-4 was performed on BMDM exposed to LPS for 24 h (Figure 1). In addition expression of ADAMs 8, 15, 17, 33, ADAMTS1 and 4 was determined as they are known to be expressed in human monocytic cells following differentiation [10] and/or have been associated with inflammation [37], [38]. Since the Raw264.7 macrophage cell line is often used as a model system for macrophages, we also assessed the response of these cells to LPS (Figure 1). Table 1 depicts those MMPs, ADAMs and TIMPs whose steady state mRNA expression was significantly regulated by LPS in these two macrophage populations (confirmed in 2 further experiments). In BMDM, expression of several MMPs was up-regulated as previously reported, including MMP9 [39], [40] and MMP14 [41]. Although MMP12 was significantly down-regulated by LPS, this proteinase was still highly expressed (data not shown). Expression of MMPs was also differentially regulated by LPS in Raw264.7 cells with several genes regulated in a similar manner to those in BMDM (MMP10, 11, 14, and 25). Some differences were observed, however. For example, whilst MMP2 was significantly up-regulated in BMDM, expression was not detected under any condition in Raw264.7 cells. Several MMPs were not expressed in either BMDM or Raw264.7 in either untreated or LPS-treated cells. Expression of ADAM8 and 15 was partially repressed in BMDM by LPS. Amongst the TIMPs only TIMP2 expression was partially but significantly repressed in both BMDM and Raw264.7 cells.

**Table 1:** The regulation of metalloproteinases and TIMPs in response to LPS.

	*BMDM*	*Raw264.7*
MMP2	↑*	–
MMP3	no effect	↑*
MMP8	no effect	↑*
MMP9	↑*	no effect
MMP10	↓***	↓***
MMP11	↓*	↓**
MMP12	↓*	no effect
MMP13	no effect	↑***
MMP14	↑***	↑**
MMP15	↓**	no effect
MMP21	–	↓*
MMP25	↑***	↑***
MMP27	↓***	–
MMP28	no effect	↑***
TIMP2	↓***	↓*
ADAM8	↓*	no effect
ADAM15	↓*	no effect

* p≤0.05.

** p≤0.01.

*** p≤0.001.

‘–’ indicates no expression.

Primary BMDM and Raw264.7 macrophages were cultured with 100 ng/mL LPS for 24 h prior to RNA extraction, reverse transcription and qRT-PCR analysis. Arrows indicate trend change of gene expression compared to untreated samples. Only expression changes of greater than 1 C_T_ are included.

### Steady-state mRNA Levels of MMP10 are Differentially Regulated by LPS Over Time and in an NF-κB-dependent Manner

**Figure 2:**
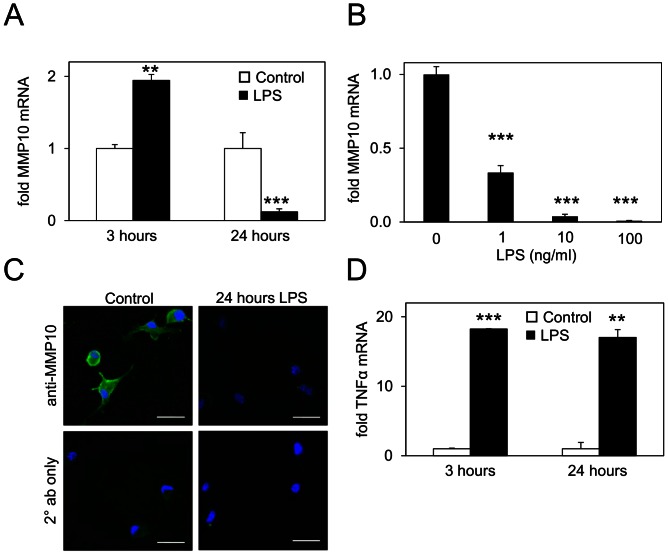
Macrophage MMP10 expression is modulated in a time-dependent manner in response to LPS. (A) Increased steady-state levels of MMP10 mRNA 3 h post-LPS treatment in BMDM and decreased levels of MMP10 mRNA 24 h post-LPS treatment as determined by qRT-PCR. (B) Repression of MMP10 mRNA expression following 24 h LPS treatment is dose-dependent. (C) Immunofluorescence reveals decreased levels of MMP10 protein (green) expressed by BMDM 24 h post-LPS treatment compared to untreated cells (all cells treated with monensin). DAPI nuclear stain is shown in blue [scale bar = 25 µm]. (D) BMDM expression of TNFα mRNA is increased 3 h post-LPS treatment and remains enhanced 24 h post-LPS treatment. All mRNA expression is normalised to 18S endogenous control. **p≤0.01, ***p≤0.001 (Student’s T test). Each bar represents mean ± S.E.M. of at least 3 samples. Experiments were performed 3 times and a representative experiment is shown.

Novel MMP regulation in BMDM by LPS included the significant repression of MMP10 at 24 h (Table 1). Given that LPS is typically regarded as an inducer of MMP expression, we explored MMP10 expression at an earlier time point. Interestingly, LPS significantly induced expression of MMP10 at 3 h (Figure 2A), with expression returning to basal levels by 8 h (Figure S1). MMP10 expression was repressed up to 7-fold below basal levels by LPS at 24 h (Figure 2A) and in a dose-dependent manner (Figure 2B). Importantly, the repression of MMP10 expression by LPS was also observed at the protein level as revealed by immunolocalisation in BMDM treated with the intracellular protein transport inhibitor, monensin, thus preventing MMP secretion (Figure 2C). We took this approach because levels of MMP10 secreted by murine BMDM are low and do not permit ready detection by Western blotting. TNFα expression was markedly induced in BMDM at 3 h and remained highly expressed at 24 h post-stimulation (Figure 2D) confirming that LPS mediated a predictable pro-inflammatory response in these cells. An extended time-course of MMP10 expression revealed that by 48 h after LPS treatment steady state mRNA levels for MMP10 had returned to basal levels (Figure S1).

**Figure 3:**
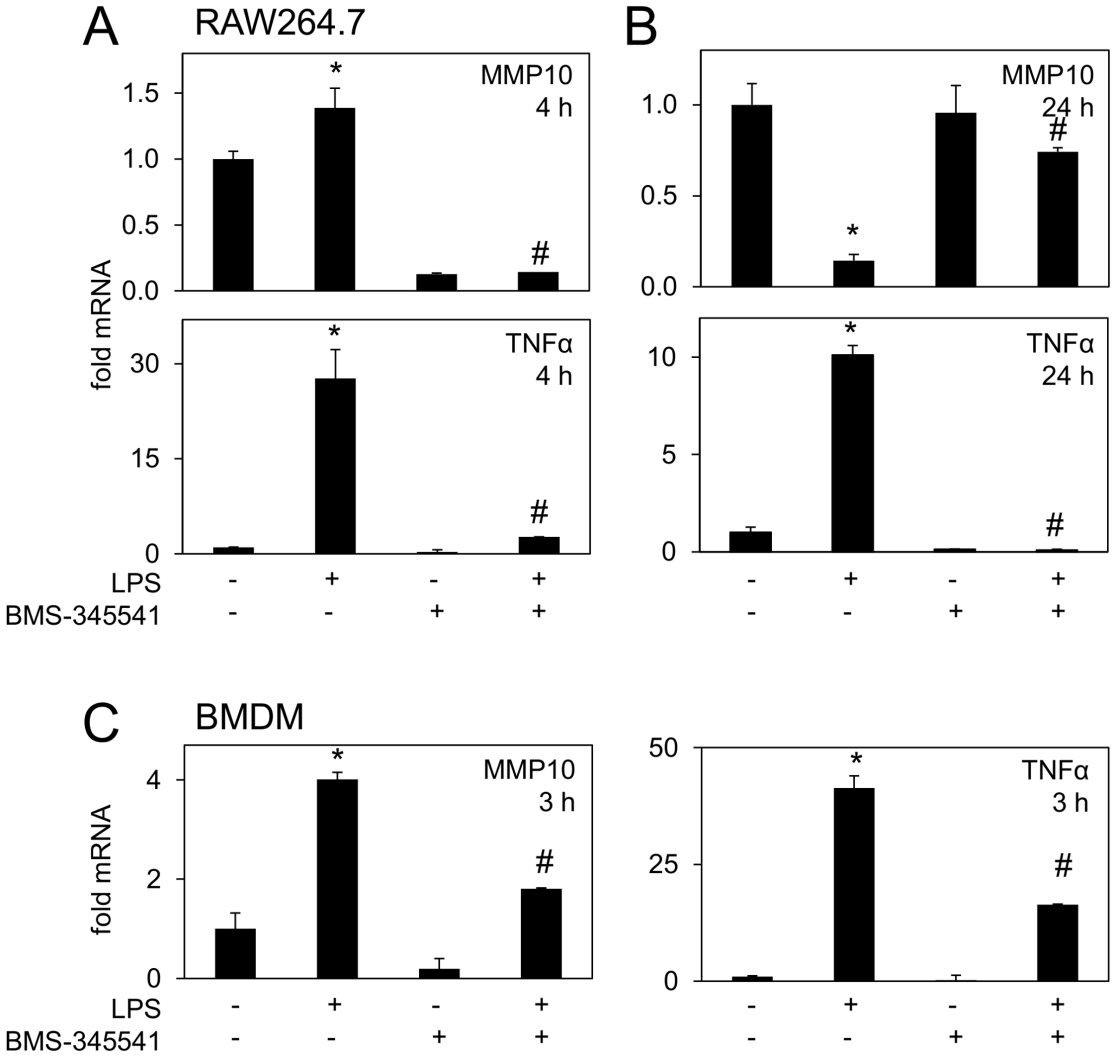
The time-dependant effects of LPS on macrophage MMP10 expression are sensitive to NF-κB inhibition. (A) Induction of MMP10 (above) and TNFα mRNA (below) in LPS-treated Raw264.7 macrophages was abrogated by IKK inhibitor BMS-345541 at 4 h as determined by qRT-PCR (B) The repression of MMP10 mRNA (above) 24 h post-LPS treatment was also reversed by BMS-345541 (below) whilst TNFα induction was still repressed (right). (C) Abrogation of MMP10 (left) and TNFα (right) expression was also observed in the presence of BMS-345541 in BMDM 3 h post-LPS exposure. *p≤0.05, relative to untreated samples, ^#^p≤0.05, relative to LPS alone (Student’s T test). Each bar represents mean ± S.E.M. of at least 3 samples. Experiments were performed 3 times and a representative experiment is shown.

To address the potential mechanisms involved in MMP10 expression in macrophages, the effect of the IκB kinase (IKK) inhibitor BMS-345541 was assessed. Inhibition of IKK repressed LPS induction of MMP10 and TNFα at 4 h in Raw264.7 cells (Figure 3A, upper and lower panels respectively) and also in BMDM at 3 h (Figure 3C). Interestingly, this inhibitor also largely reversed the LPS-mediated repression of MMP10 at 24 h in Raw264.7 cells (Figure 3B, upper panel) suggesting that LPS regulates MMP10 expression via an NF-κB-dependent mechanism. However, endogenous expression of MMP10 at 24 h was not inhibited by the IKK inhibitor (Figure 3B, upper panel) suggesting that other signalling pathways must be involved. TNFα induction in Raw264.7 cells was also repressed at 24 h (Figure 3B, lower panel). BMS-345541 proved toxic to BMDM at 24 h such that no data could be generated. Taken together these data suggest that the NF-kB pathway is involved in both the induction and repression of MMP10 by LPS.

### BMDM Migrate Across Several ECM Substrates Showing a Preference for Fibronectin

**Figure 4:**
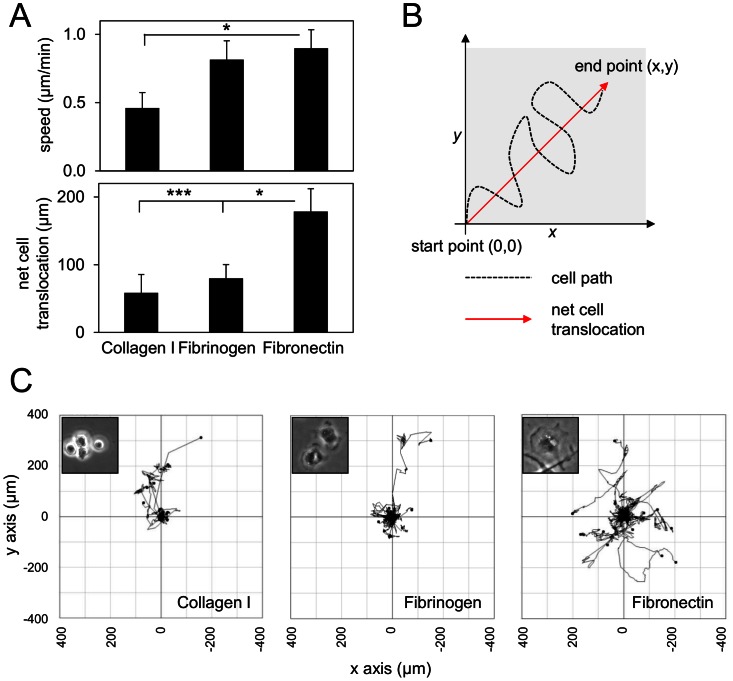
Fibronectin is a good substrate for random migration of primary macrophages. BMDM were cultured on thin coatings of collagen I, fibrinogen or fibronectin for 24 h. (A) Time-lapse microscopy analysis reveals no significant variation in speed of macrophage migration (above), however net cell translocation (below), is significantly enhanced on fibronectin when compared to other conditions. *p≤0.05, ***p≤0.001 relative to fibronectin (Student’s T test). Each bar represents mean ± S.E.M. of at least 10 cell tracks. (B) Schematic explanation of net cell translocation by cells. (C) Migration plots illustrate the enhanced motility of macrophages on fibronectin. Insets: stills from time-lapse recordings. Note cells on collagen I remain rounded. Experiments were performed 3 times and a representative experiment is shown.

As MMP10 has been suggested to affect migration of other cell types [42], we hypothesised that its endogenous expression may play a similar role in BMDM motility. We first determined an optimal substrate for these cells in 2D time-lapse random migration assays, selecting three substrates of relevance to macrophage biology. A 2D substrate of fibronectin resulted in the highest BMDM random migration speed (Figure 4A, left panel), similar to that observed over fibrinogen. Macrophages migrating over fibronectin showed significantly enhanced net cell translocation (Figure 4B) compared to either collagen I or fibrinogen (Figure 4A, upper panel). Corresponding plots of BMDM migration reflect these observations (Figure 4C). Collagen I was a poor substrate for macrophage adhesion, failing to induce cell spreading (inset Figure 4C left panel) although supporting migration of the still-rounded cells (Figure 4C left panel).

### Gene Silencing of Endogenous MMP10 Represses Random Migration of BMDM

**Figure 5:**
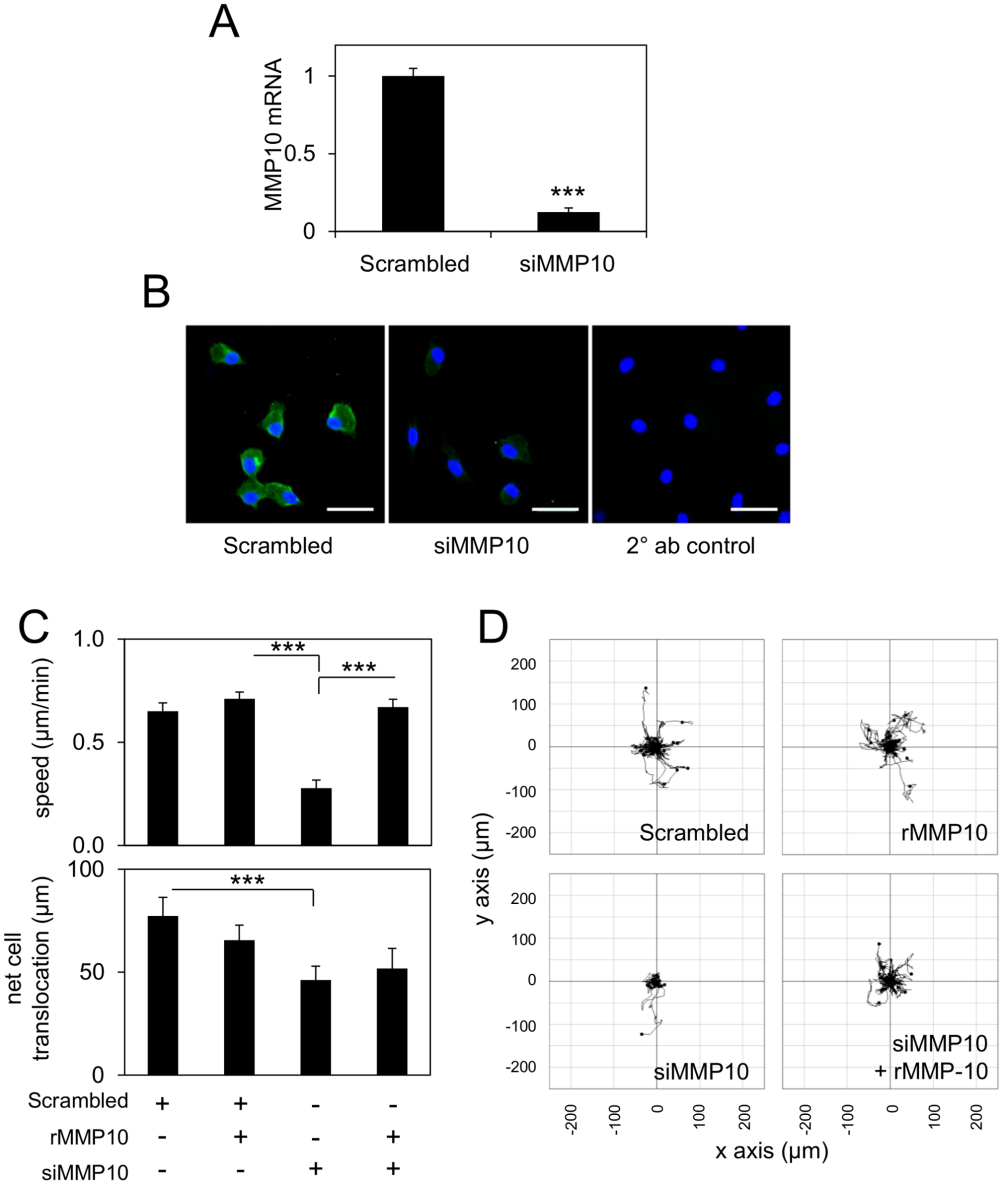
Repression of random macrophage migration on fibronectin by gene silencing of MMP10 and rescue by the exogenous application of soluble rMMP10. (A) qRT-PCR analysis reveals successful repression of MMP10 mRNA expression in BMDM transfected with siMMP10 compared to scrambled control siRNA. ***p≤0.001. Each bar represents mean ± S.E.M. of at least 3 samples. (B) MMP10 protein immunolocalisation in BMDM 24 h post-transfection with siMMP10 shows repression of protein levels compared to scrambled control siRNA and secondary (2°) antibody only control, (all cells treated with monenesin), [scale bar = 25 µm]. (C) Time-lapse microscopy analysis reveals a significant reduction in the speed of BMDM migration (above) on fibronectin following siMMP10 transfection, which was rescued by exogenous rMMP10. Analysis of net cell translocation (below) confirms the siMMP10 driven reduction in migration. (D) Corresponding migration plots reflect the effect of siMMP10 and rMMP10 on macrophage motility. ***p≤0.001 (Student’s T test). Each bar represents mean ± S.E.M. of at least 10 cell tracks. Experiments were performed 3 times and a representative experiment is shown.

To explore the role of endogenously expressed MMP10 in BMDM migration, we used a gene silencing approach, transfecting BMDM with siRNA targeting MMP10 (siMMP10), which was verified at both mRNA (Figure 5A) and protein levels (Figure 5B) by immunofluorescence of monensin-treated cells. Transfection of BMDM with siMMP10 resulted in an approximately 3-fold reduction in cell migration speed and a significant reduction in net cell translocation compared to scrambled siRNA transfection (Figure 5C, upper and lower panels; corresponding cell tracks in Figure 5D). Importantly, the reduction in migration speed driven by siMMP10 could be rescued by the addition of exogenous rMMP10 protein (Figure 5C, D). Interestingly, the further addition of rMMP10 to scrambled siRNA-transfected cells did not enhance BMDM speed any further, suggesting that the endogenous level of MMP10 is sufficient to produce the maximum migration effect under these conditions. These results demonstrate that endogenous MMP10 expression plays a role in macrophage migration over a fibronectin substrate.

### BMDM from *Mmp10^−/−^* mice have Substantially Impaired Migration Over a Fibronectin Substrate

**Figure 6:**
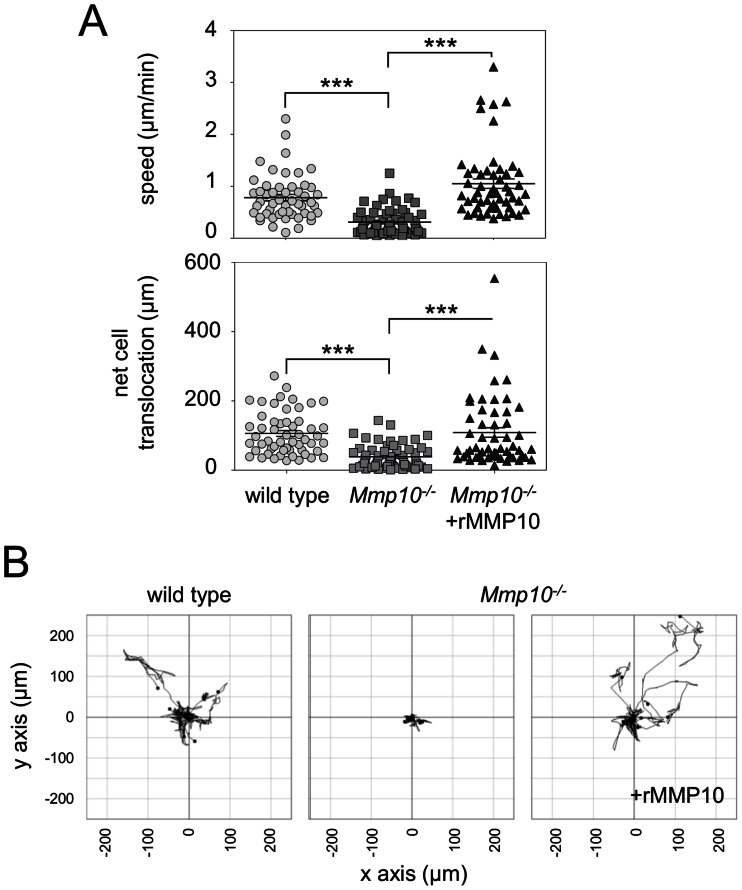
*Mmp10^−/−^* macrophages display reduced migration that can be rescued with the exogenous application of soluble rMMP10. BMDM from the *Mmp10^−/−^* mouse and their back-crossed wild-type (WT) litter-mates were cultured on fibronectin for 24 h before the addition of rMMP10 as indicated. (A) Time-lapse microscopy analysis of *Mmp10^−/−^* BMDM (squares) reveals significantly reduced migration speed (above) and net cell translocation distance (below) compared to wild-type BMDM (circles). Exogenous application of rMMP10 to the culture medium of *Mmp10^−/−^* BMDM (triangles) enhances migration speed (above) and net cell translocation (below) to levels comparable to wild-type BMDM (circles). (B) Examples of migration plots reflect the effect on BMDM motility. ***p≤0.00.1 (Student’s T test). Data depicts mean ± S.E.M. of at least 60 cell tracks in combined data from cells isolated in two independent experiments (2 mice per genotype).

To further substantiate a role for MMP10 in BMDM migration, similar 2D migration assays were performed on a fibronectin substrate with BMDM differentiated from the bone marrow of MMP10 null (*Mmp10^−/−^*) mice. *Mmp10^−/−^* BMDM showed a significant 2-fold reduction in cell migration speed (0.31+/−0.03 µm/h compared to 0.7+/−0.05 µm/h for wild-type cells) as well as a significant reduction in net cell translocation compared to wild-type littermate-derived BMDM (Figure 6A, upper and lower panels). The migration deficit observed in *Mmp10^−/−^* BMDM was rescued by the addition of rMMP10 (0.96+/−0.08 µm/h) and examples of cell tracks of BMDM migration are depicted in Figure 6B.

### MMP10 is Necessary for Invasion of BMDM into a Reconstituted 3D ECM Matrix

**Figure 7:**
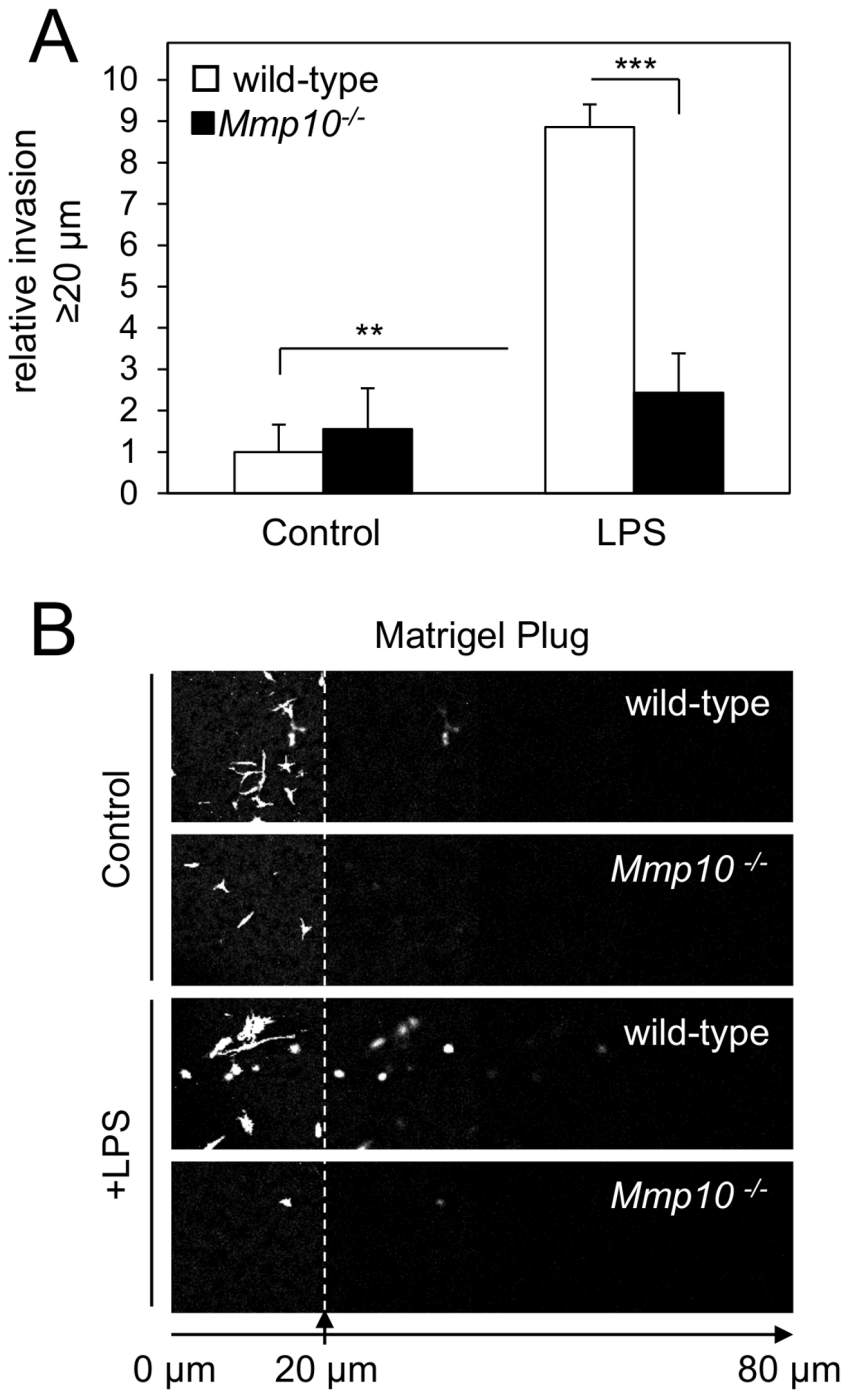
Invasion through a 3D Matrigel/fibronectin gel is impaired in *Mmp10^−/−^* macrophages. An inverted invasion assay was performed to compare 3D invasion of BMDM from the *Mmp10^−/−^* mouse and their back-crossed wild-type litter-mates. BMDM were allowed to invade into a Matrigel/fibronectin gel for 72 h along a gradient of LPS. (A) Relative invasion was quantified by measuring the fluorescence intensity of Calcein-AM staining in BMDM penetrating ≥20 µm into the gel as a percentage of the total fluorescence intensity of all cells within the gel, and is expressed relative to untreated wild-type BMDM invasion. **p≤0.01, ***p≤0.001. Each bar represents mean± S.E.M. for combined data collected from a total of 12 fields of view (3 per gel) for cells isolated independently from 2 mice per genotype. (B) Invading wild-type and *Mmp10^−/−^* BMDM were stained with Calcein-AM and visualised by confocal microscopy. Serial optical sections were captured at 20 µm intervals and typical examples, with and without LPS gradient, are presented in sequence, left to right.

Since macrophages migrate over 2D surfaces, such as the endothelium, but also through 3D matrices, such as the basement membrane, the ability of BMDM to invade a thick layer of Matrigel, supplemented with fibronectin, was explored. In these experiments we used LPS as a chemoattractant, but since the end-point of our experiments was at 72 h any effect of LPS on MMP10 expression will have subsided, since MMP10 expression returns to basal levels by 48 h (Figure S1). A gradient of LPS induced wild-type BMDM invasion whilst *Mmp10^−/−^* BMDM showed a 3-fold reduced ability to invade (Figure 7A; representative montages of confocal sections shown in Figure 7B). Importantly approximately similar numbers of *Mmp10^−/−^* BMDM and their wild-type counterparts were apparent on the underside of the filter as assessed at the end-point of the experiment, prior to imaging invasion (Figure S2). These results suggest that MMP10 plays a key role in macrophage invasion into a 3D matrix.

## Discussion

Macrophage migration is implicated in both normal and pathological conditions: it is essential for wound healing and immune responses but is an unwanted response in inflammatory conditions such as atherosclerosis and chronic wounds. In this study a comprehensive survey of metalloproteinase expression has revealed novel regulation of MMP10 in response to LPS. Further study uncovered a functional role for this metalloproteinase in the migration of macrophages on 2D components of ECM as well as in 3D invasion.

### Regulation of Metalloproteinase Expression in BMDM and Raw264.7 Macrophages by LPS

Comparison of BMDM and the Raw264.7 macrophage cell line response to LPS stimulation for 24h revealed that although some genes were differentially modulated in the two cell populations, others were significantly regulated in a similar manner. For example; up-regulation of expression of MMP14 and 25 was observed in both populations whereas that of MMP2 and 9 was induced only in BMDM. Regulation of MMP14 was in agreement with previous observations in human monocytes [8], [43]. MMP14 has been implicated in the migration of human monocytes in response to chemoattractant stimuli on a number of substrates, including fibronectin [23]. MMP25 (MT3-MMP) has been shown previously to be up-regulated in macrophages of atherosclerotic plaques, indicating a potential inflammatory association [11] and was here found to be significantly up-regulated in both Raw 264.7 macrophages and BMDM in response to LPS. Expression of several metalloproteinases was partially down-regulated in BMDM following exposure to LPS for 24 h. These include MMP12, implicated in macrophage migration [4], and MMP15 (MT2-MMP), whose function in macrophages remains to be established.

The expression of MMP10 in response to LPS was of great interest since this proteinase seems to be increasingly associated with inflammation in a variety of physiological and pathological conditions. Induction of MMP10 by LPS in primary human monocytes has been shown previously at 4 h [44] and we have confirmed these results in murine BMDM and Raw264.7 cells. TNFα was elevated more than 10-fold at 3 h after LPS stimulation and others have demonstrated substantial secretion of this cytokine from macrophages within 4 h [45]. TNFα may be responsible for MMP10 induction in this early time-frame, since TNFα can induce some MMP genes within one hour of stimulation, at least in other cell types [46]. In our study, treatment with an IKK inhibitor suppressed both endogenous and induced MMP10 and TNFα at 4 h in macrophages, supportive of a role for the NF-κB pathway in MMP10 expression/up-regulation. Recently, Huang and colleagues [47] have demonstrated that LPS/IFNγ treatment leads to induction of MMP10 expression in human monocytes which is mediated by several pathways, including NF-κB. These authors also co-localised MMP10 with NF-κB in macrophage-rich areas of human atherosclerotic plaques, lending support to a role for NF-κB in MMP10 regulation *in vivo*. Previous studies have shown that TNFα induces MMP10 expression in intestinal epithelial cells [48] and MMP10 expression in chondrocytes is also repressed by IKKβ inhibition [49]. MMP10 is induced in the tracheal epithelium of mice infected with the gram-negative bacterium *P. aeruginosa,* and mediates a range of transcriptional responses. Furthermore, analysis of known key gene interaction networks involved in host response to infection with *P. aeruginosa* suggested a role for NF-κB in the regulation this MMP [15]. Overall, our data are in keeping with the idea that the up-regulation of MMP10 by LPS at 4 h may be mediated through an NF-κB-dependent mechanism, and it has previously been shown that TNFα-induced MMP10 expression is Rel A/p65 dependent [50]. However, further investigation is needed to substantiate the involvement of this pathway in our system.

### Potential Mechanisms Underpinning Repression of MMP10 Expression

At 24 h following LPS treatment steady-state mRNA levels for MMP10 were substantially repressed 7-fold below basal levels with very little expression observed at the protein level at this time-point. It should be noted that others have observed up-regulation of MMP10 expression by LPS treatment in human monocytes at 18 h [8], which may be a reflection of the shorter time of exposure and/or species. It is possible that in human monocytes MMP10 expression maintains a prolonged elevation. It is of interest to note that, whilst at 24 h expression of MMP10 is suppressed, TNFα expression remains high, indicating that this cytokine is likely not implicated in regulating MMP10 expression at this later time-point. The time-dependent repression of MMP10 expression following LPS treatment we observed in both BMDM and Raw264.7 macrophages may reflect the effect of several signalling pathways. Interestingly pharmacological inhibition of IKK largely reversed LPS-driven differential MMP10 expression, indicating NF-κB pathway involvement. The potential role of NF-κB in MMP10 repression is further supported by the elegant study of Treiber et al. [51], who have demonstrated that LPS treatment of BMDM from mice with functionally inactive RelA/p65 (resulting in abrogation of NFKB signalling) results in up-regulation of MMP10 expression, in keeping with our observations at 24 h post-LPS treatment.

Epigenetic mechanisms of MMP10 regulation have also been reported with histone deacetylase (HDAC)7 shown to repress MMP10 expression in endothelial cells through sequestration of the transcription factor MEF2 [52]. In addition, under TNFα stimulation HDAC7 dissociates from MEF2 making MEF2 available, allowing subsequent transcription of MMP10 [53]. LPS up-regulates expression of MEF2 in monocytes [54] as well as HDAC7 in BMDM [55], which could result in overall MMP10 suppression depending on relative levels of these proteins. In our hands we did not observe modulation of HDAC7 expression at the mRNA level although we did observe repression of HDAC5 by LPS (data not shown) and it is noteworthy that HDAC5 can also bind to and regulate expression of MEF2 (reviewed in [56]). However given that HDAC7 and MEF2 [54] are also regulated through phosphorylation events, mRNA levels of HDAC7 may not be as important as protein localisation, following LPS treatment. Zinc finger protein 267 (ZNF267) has also been reported to act as a transcriptional repressor of MMP10 expression in hepatic stellate cells, in an HDAC-independent manner [57]. However, these authors commented that LPS does not transactivate ZF267, thus making it less likely to be involved in our system. MicroRNAs are implicated in repression of many genes and a candidate in our system may be miR-155 since this microRNA has been shown to be involved in LPS regulation of several pathways in dendritic cells [58]. Future studies will determine whether these pathways are involved in the LPS-mediated repression of MMP10.

### MMP10 Regulation of Cell Migration

In both 2D time-lapse migration assays and in 3D invasion we have shown that MMP10 is a key player in macrophage migration. Whilst MMP10 has not previously been studied in macrophage migration, its exogenous expression has been associated with migrating keratinocytes in wound healing with MMP10 inducing keratinocyte migration *in vitro* [42]. MMP10 is regulated in a spatiotemporal manner during wound healing, with strong expression at both day 1 and day 5 post-wounding [59] and has been observed in macrophage-rich areas of the dermis in human skin ulcers [60]. We have similarly localised MMP10 in macrophage-rich areas of granulation tissue of mouse skin wounds (Murray, Bevan and Gavrilovic unpublished observations). Of interest, circulating MMP10 levels have been correlated with markers of inflammation and increased atherosclerotic plaques in patients with elevated cardiovascular disease risk [61]. In addition, circulating levels of MMP10 are significantly higher in patients with sepsis [62], suggesting an important role for this proteinase during infection.

Current concepts of leukocyte cell migration and cell-matrix interactions have been established in 2D models [63], [64], [65]. Leukocytes adhere to the undulating, 2D, layer of activated endothelium lining the blood vessel lumen; rolling then crawling over it, before subsequent diapedesis and invasion through the basement membrane and underlying stroma (reviewed in [66], [67]). LPS has been shown to act as a chemo-attractant for macrophages [68] and we confirm here the ability of LPS to induce macrophage invasion when applied across an ECM gel. The ability of LPS to induce invasion may seem contradictory given our observations regarding MMP10 suppression, however, the steady-state levels of MMP10 have returned to basal levels by 48 h post-LPS exposure and given our invasion assay end point is at 72 h any suppressive role of LPS will have long since subsided.

Our experiments thus highlight a central role for metalloproteinases, with both 2D migration and 3D invasion of BMDM found to be MMP10-dependent. Mechanistically, migration and invasion have been shown to have several different characteristics (reviewed in [69]) and invasion of some cell lines through Matrigel has been shown by others to be independent of proteolytic activity [70]. However, it is possible that MMP10 cleaves the fibronectin substrate present in our model, and/or cell surface molecules such as syndecans, or endogenous chemokines, as reported for other MMPs (reviewed in [3]). Importantly, others have shown that cysteine proteinases, including cathepsin B, can mediate macrophage invasion [71], [72] suggesting that proteinase involvement may be context- and stimulus-dependent.

In conclusion our studies demonstrate a key role for MMP10 in both 2D and 3D migratory contexts and future studies will uncover the precise roles played by this metalloproteinase, which is of emerging importance in macrophage biology.

## Supporting Information

Figure S1 **An extended time course of macrophage MMP10 expression following treatment with LPS.** Steady state mRNA levels for MMP10 are elevated at 4 h, return to basal levels by 8 h and are repressed at 24 h. By 48 h MMP10 expression levels return to untreated levels and a similar level of expression is observed at 72 h.
(TIF)

Figure S2 **Typical examples to demonstrate approximately equal numbers of wild-type and** ***Mmp10^−/−^*** **BMDM adhering to the lower surface of the Transwell™ filters for the inverted invasion assay.** BMDM were stained with Calcein-AM and visualised by confocal microscopy.
(TIF)
